# Teriparatide Induced Delayed Persistent Hypercalcemia

**DOI:** 10.1155/2014/802473

**Published:** 2014-08-18

**Authors:** Nirosshan Thiruchelvam, Jaskirat Randhawa, Happy Sadiek, Gaurav Kistangari

**Affiliations:** ^1^Internal Medicine Residency, Fairview/Cleveland Clinic Foundation, Cleveland, OH 44111, USA; ^2^Department of Hospital Medicine, Cleveland Clinic Foundation, Cleveland, OH 44195, USA

## Abstract

Teriparatide, a recombinant PTH, is an anabolic treatment for osteoporosis that increases bone density. Transient hypercalcemia is a reported side effect of teriparatide that is seen few hours following administration of teriparatide and resolves usually within 16 hours of drug administration. Persistent hypercalcemia, although not observed in clinical trials, is rarely reported. The current case describes a rare complication of teriparatide induced delayed persistent hypercalcemia.

## 1. Introduction

Teriparatide, a recombinant human parathyroid hormone (PTH), is currently the only FDA approved anabolic agent in the United States for the treatment of osteoporosis [1]. It is indicated for patients with severe osteoporosis who are at high risk of vertebral and nonvertebral fractures as well as for those with glucocorticoid induced osteoporosis [2, 3]. The commonly reported side effects of teriparatide include nausea, vomiting (8.6%), hypertension (5.2%), dizziness (4.1%), and allergic reactions (3.6%) [4]. Teriparatide induced transient hypercalcemia is frequently observed between 4 and 6 hours following dose and often returns to baseline by 16 hours following drug administration [5, 6]. We report a patient who had persistent hypercalcemia that required aggressive in-hospital treatment. This case also underlines the importance of monitoring serum calcium level in certain high risk patients while on teriparatide.

## 2. Case Presentation

**Figure 1:**
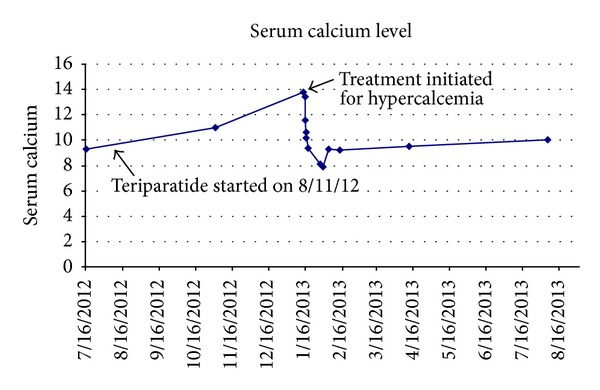


A 65-year-old woman presented to her primary care physician for a preoperative evaluation for total hip arthroplasty. She was found to have incidental calcium level of 13.8 mg/dL (normal range (NR), 8.5–10.5 mg/dL) on routine laboratory evaluation. Her past medical history was significant for steroid dependent COPD, hypothyroidism, severe scoliosis with lumbar spinal stenosis, severe degenerative osteoarthritis of the left hip, and severe osteoporotic changes in lower thoracic and lumbar region (bone mineral density, 4.9). For the latter condition, she has been on teriparatide 20 mcg subcutaneous injection daily for 5 months, after failing to respond to bisphosphonate therapy. Her other medications included levothyroxine 25 mcg, prednisone 20 mg, 1000 units of Vitamin D, and 1000 mg of calcium supplementation daily. On further questioning, she reported increased thirst, constipation, and nausea for one month. Her vital signs were stable and physical examination was unremarkable. Results of the initial blood tests revealed hemoglobin 12.1 g/dL (NR, 13–17 g/dL), mean corpuscular volume 96.5/fL (NR, 80–100 fL), white blood cell 8800/mL (NR, 4000–11000/mL), platelet count 288 000/mL (NR, 150 000–400 000/mL), calcium level 13.8 mg/dL (NR, 8.5–10.5 mg/dL, drawn 24 hours after last dose of teriparatide), creatinine level 0.73 mg/dL (0.60–1.00 mg/dL), and blood urea nitrogen level 18 mg/dL (NR, 8–25 mg/dL). Prior to the initiation of teriparatide, her calcium level was 9.3 mg/dL (Figure 1). Patient was subsequently admitted to the hospital for the management of hypercalcaemia. She was treated with intravenous fluids using 0.9% saline (3 liters initial bolus) followed by slow continuous intravenous furosemide for 16 hours. However, patient only responded to the above treatment partially. She was then treated with calcitonin (100 units, 2 doses) and intravenous pamidronate (30 mg). Following this, after 33 hours, patient's fatigue improved and her serum calcium level decreased to 11.6 mg/dL. Her calcium level was monitored that eventually returned to normal range after 48 hours (Figure 1).

Other work-up included PTH < 4 pg/mL (NR, 10–60 pg/mL), vitamin D 25 hydroxy 47.1 ng/mL (NR, 31–80 ng/mL), vitamin D 1,25 dihydroxy 4 pg/mL (NR, 15–60 pg/mL), phosphorus 3.7 mg/dL (NR, 2.5–4.5 mg/dL), magnesium 1.5 mg/dL (NR, 1.7–2.6 mg/dL), ESR 22 mm/hr (NR, 0–30 mm/hr), CRP 1.1 mg/dL (NR, 0.0–1.0 mg/dL), parathyroid-related peptide 0.3 pmol/L (NR, <2 pmol/L), and TSH 1.84 *μ*U/mL (NR, 0.400–5.00 *μ*U/mL). Her serum protein electrophoresis and skeletal survey were normal. 

At the time of discharge, her PTH returned to 16 pg/mL. Sheremained off teriparatide treatment and continued to be on the same dose of calcium and vitamin D supplementation. Her serum calcium remained normal for the rest of her follow-up. She also had normal 24-hour urinary calcium level which apparently excluded the possibility of familial hypocalciuric hypercalcemia, 294.5 mg/24 hr (NR, 100–300 mg/24 hr).

## 3. Discussion

Osteoporosis is a major worldwide health problem [7]. Teriparatide has been approved for the treatment of osteoporosis in postmenopausal women at high risk for vertebral and nonvertebral fractures. The recommended duration of teriparatide therapy is 2 years in the United States and 18 months in Europe. There is lack of safety and efficacy of the drug beyond 2 years in available clinical trials [8]. The effects of teriparatide have been studied in postmenopausal women and in men with advanced osteoporosis. In a study of postmenopausal women, teriparatide was administered as a 20 *μ*g daily subcutaneous injection, and the author demonstrated an increase in vertebral bone mineral density (BMD) by 8 to 9% and femoral BMD by about 3% over a 21-month period. It was also associated with 54% reduction in the fracture incidence at nonvertebral sites and a 65% reduction in fracture incidence at vertebral sites [8].

Adverse events with teriparatide include mild hypercalcemia, which has been reported in 1 to 3% of patients [9]. Teriparatide is both rapidly absorbed and eliminated with a short half-life of 1 hour. Due to its pharmacokinetic profile, which causes the drug to reach peak level rapidly within few hours of administration, a transient elevation of calcium is observed 4–6 hours after injection, and this is on average a small effect. The maximum postdose (4–6 hours after dosing) serum calcium in the phase-3 trial was about 11 mg/dL [5, 6]. Serum calcium concentrations returned to baseline by 16 hours following dose. Patients in clinical trials did not require treatment for hypercalcemia [5]. In a large phase-3 clinical trial, none of the patients had serum calcium > 11 mg/dL, which was measured approximately a day after dosing [5]. In postmarketing surveillance, some patients have had hypercalcemia with the highest reported values > 13 mg/dL, but further information is lacking on these patients [6]. Our patient had hypercalcaemia that persisted more than 33 hours even after aggressive fluid resuscitation treatment. As of our knowledge, this is the third reported case in the literature with persistent hypercalcemia secondary to teriparatide therapy [10, 11]. Few strategies that may be effective in preventing hypercalcemia include reducing calcium supplementation and dosage adjustment of teriparatide from daily to every-other-day administration [8]. It may perhaps be reasonable to monitor serum calcium levels in such patients while further studies with more follow-up periods are available. Transient hypercalcemia is a well-known adverse effect which usually subsides by 16 hours of teriparatide administration. Persistent hypercalcemia could be seen as a rare side effect of teriparatide treatment. Patients at risk of developing hypercalcemia should be monitored closely while on treatment with teriparatide.

## References

[1] Augustine M, Horwitz MJ (2013). Parathyroid hormone and parathyroid hormone-related protein analogs as therapies for osteoporosis. *Current Osteoporosis Reports*.

[2] Christenson ES, Jiang X, Kagan R, Schnatz PF (2012). Osteoporosis management in post-menopausal women. *Minerva Ginecologica*.

[3] Mazziotti G, Angeli A, Bilezikian JP, Canalis E, Giustina A (2006). Glucocorticoid-induced osteoporosis: an update. *Trends in Endocrinology & Metabolism*.

[4] Migliaccio S, Resmini G, Buffa A (2013). Evaluation of persistence and adherence to teriparatide treatment in patients affected by severe osteoporosis (PATT): a multicenter observational real life study. *Clinical Cases in Mineral and Bone Metabolism*.

[5] Krege JH, Glass E, Donley D Teriparatide treatment was not associated with predosehypercalcemia in treatment-naive women with osteporosois.

[6] Eli Lilly and Company http://pi.lilly.com/us/forteo-pi.pdf.

[7] Looker AC, Orwoll ES, Johnston CC (1997). Prevalence of low femoral bone density in older U.S. adults from NHANES III. *Journal of Bone and Mineral Research*.

[8] Canalis E, Giustina A, Bilezikian JP (2007). Mechanisms of anabolic therapies for osteoporosis. *The New England Journal of Medicine*.

[9] Gold DT, Pantos BS, Masica DN, Misurski DA, Marcus R (2006). Initial experience with teriparatide in the United States. *Current Medical Research and Opinion*.

[10] Hajime M, Okada Y, Mori H, Tanaka Y (2014). A case of teriparatide-induced severe hypophosphatemia and hypercalcemia. *Journal of Bone and Mineral Metabolism*.

[11] Karatoprak C, Kayatas K, Kilicaslan H (2012). Severe hypercalcemia due to teriparatide. *Indian Journal of Pharmacology*.

